# Applications of Surface Plasmon Resonance in Heparan Sulfate Interactome Research

**DOI:** 10.3390/biomedicines13061471

**Published:** 2025-06-14

**Authors:** Payel Datta, Jonathan S. Dordick, Fuming Zhang

**Affiliations:** 1 Department of Life Sciences, Albany College of Pharmacy and Health Sciences, Albany, NY 12208, USA; payel.datta@acphs.edu; 2 Department of Chemical and Biological Engineering, Center for Biotechnology and Interdisciplinary Studies, Rensselaer Polytechnic Institute, Troy, NY 12180, USA

**Keywords:** surface plasmon resonance, heparan sulfate, heparin, interactome

## Abstract

Surface plasmon resonance (SPR) is a powerful tool for analyzing biomolecular interactions and is widely used in basic biomedical research and drug discovery. Heparan sulfate (HS) is a linear complex polysaccharide and a key component of the extracellular matrix and cell surfaces. HS plays a pivotal role in maintaining cellular functions and tissue homeostasis by interacting with numerous proteins, making it essential for normal physiological processes and disease states. Deciphering the interactome of HS unlocks the mechanisms underlying its biological functions and the potential for novel HS-related therapeutics. This review presents an overview of the recent advances in the application of SPR technology to HS interactome research. We discuss methodological developments, emerging trends, and key findings that illustrate how SPR is expanding our knowledge of HS-mediated molecular interactions. Additionally, we highlight the potential of SPR-based approaches in identifying novel therapeutic targets and developing HS-mimetic drugs, thereby opening new avenues for intervention in HS-related diseases.

## 1. Introduction

Surface plasmon resonance (SPR) is a powerful and label-free optical sensing technique used to monitor molecular interactions in real time. Because of its sensitivity and real-time monitoring capabilities, SPR has become a valuable tool in basic biomedical research, drug discovery, environmental monitoring, and bioprocessing [1,2,3,4,5,6,7,8]. SPR has been successfully utilized to investigate glycan interactions (binding kinetics and affinities) with known and potential ligands [9,10,11,12].

Heparan sulfate (HS) belongs to a family of negatively charged glycosaminoglycans and shares its biosynthetic pathway with heparin, a naturally occurring anticoagulant drug. HS and heparin are composed of repeating disaccharide units consisting of glucosamine (*N*-acetylated or *N*-sulfated) and iduronic acid or glucuronic acid. The disaccharide moieties of HS exhibit variable sulfation patterns with ~0.8 sulfate groups per disaccharide and include a combination of 6-*O*-sulfated, 2-*O*-sulfated, and *N*-sulfated moieties. The disaccharide moieties of the heparin exhibit complete modifications and contain ~2.3 sulfate groups per disaccharide, including complete 6-*O*-sulfated, 2-*O*-sulfated, and *N*-sulfated moieties. HS and heparin are covalently linked to core proteins to form heparan sulfate proteoglycans (HSPGs), which are categorized based on their presence in the cell, namely, cell surface HSPGs (glypicans and syndecans), basement membrane HSPGs (agrin, collagen type XVIII, and perlecan), and secretory granule HSPGs (serglycin). Both HS and HSPGs are present as part of the cell’s glycocalyx and have multiple essential roles in cell signaling, transduction, homeostasis, cell–cell interactions, and cell–environment interactions.

The interactome is a comprehensive representation of the biomolecular interactions within a biological system (such as a cell or organism). The HS interactome includes interactions of heparin and HS with a wide array of proteins, including extracellular matrix (ECM) proteins, lipoproteins, chemokines, growth factors, and serpins. Many excellent review articles on HS and the heparin interactome have been published [13,14,15,16]. Table 1 summarizes examples of HS and heparin interacting with various biomolecules. These interactions are essential for modulating biological functions, including cell signaling, ECM assembly, homeostasis, growth factor signaling, pathogenesis, inflammation, and cancer. The HS interactome enables a systematic study of HS-binding biomolecules (e.g., HS-binding proteins). The interactome also emphasizes the impact of modifications of the HS biosynthesis on the biological systems. A comprehensive knowledge of the HS interactome will provide a fundamental insight into the biology of the cell. In addition, the HS interactome applications also include screening of druggable HS/HP–protein interactions for therapeutic potential. Herein, we present a comprehensive overview of the applications of SPR in the study of the HS interactome.

## 2. Heparan Sulfate (HS)–Heparan Sulfate-Binding Protein (HSBP) Interactome

**Figure 1:**
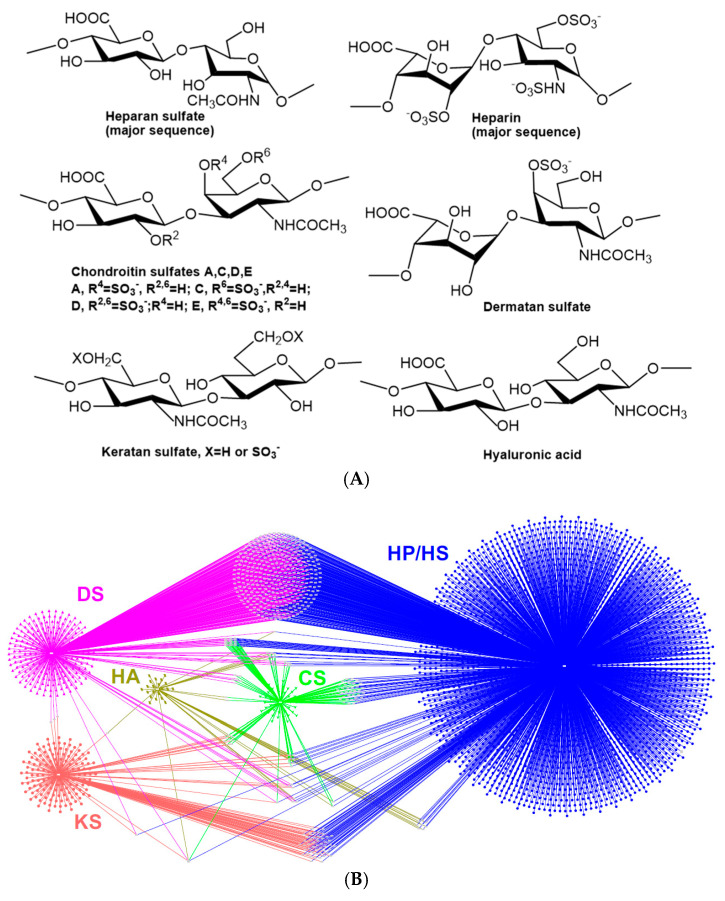
(**A**) Disaccharide structures of major classes of GAGs. (**B**) The protein–GAGs interactome network, adapted from “The GAGs interactome 2.0” with permission. HP/HS, heparin/heparan sulfate (blue); CS, chondroitin sulfate (green); DS, dermatan sulfate (pink); HA, hyaluronic acid (dark yellow); KS, keratan sulfate (red). Each dot represents one protein binding partner. Some proteins (dots in light gray) can interact with multiple GAGs.

HS/HSPGs are present as part of the cell’s glycocalyx and form a major component of the cell’s extracellular matrix. HSPGs play major roles in modulating cell signaling and homeostasis through the interaction with growth factors, growth factor receptors, chemokines, serpins, proteases, cell adhesion molecules, amyloid proteins, and lipid and membrane-binding molecules [16]. Recently, the 2nd version of the comprehensive draft of the HS/heparin interactome (including other GAG interactomes) (Figure 1) was reported by Vallet et al. (2022) and analyzed 3464 unique GAG-binding proteins and 4290 GAG–protein interactions, with 2873 for HS/heparin–HSBP interactions [44]. The comprehensive network of HS–protein interactions (Figure 1) provides a snapshot into the biological functions of HS. The authors have reiterated that the major interaction between HS–HSBP is through the Cardin–Weintraub motifs characterized by arginine/lysine residues [40]. These amino acid sequences fold to form a 3D arrangement on the HSBP surface. The 3D arrangement facilitates the binding of the HSBP to HS/heparin. The HS/heparin chain composition, including sulfated domains, non-sulfated domains, and the presence of iduronic acid moieties, impacts the conformational flexibility of the chain. The flexibility of the chain enables multiple interaction loci along the HS chain, resulting in a unique biomolecular interaction [44].

Cell surface HSPGs are involved in ligand–receptor clustering and cell signaling [45]. For example, HSPGs interact with fibroblast growth factors (FGF) and their canonical receptor (FGFR) to form an active ternary stable complex, and this interaction results in a cascading reaction that leads to influencing the cell’s physiological processes [45]. The interaction of HS and FGF is achieved through specific protein-binding domains in the HS backbone [45]. The protein-binding domains within the HS chain are formed due to the sulfation pattern of the disaccharide repeats of (GlcA/IdoAβ1-4GlcNAcα1-4)_n_. HSPGs also interact with other growth factors, including vascular endothelial growth factors (VEGF), transforming growth factor β (TGF-β), and platelet-derived growth factor (PDGF) [45]. The interaction of HSPGs with growth factors and growth factor receptors leads to activation of cell growth and cell differentiation and can also be a key player in cancer progression. It has been shown that the HS composition of tumor cells and tumor microenvironments is modified, suggesting that HS composition is one of the key players in cancer cell physiology [46,47]. For example, dysregulation of HS biosynthetic and modification enzymes (sulfotransferases and sulfatases) correlates with tumor metastasis [46].

Serpins are a family of proteins that share structural and functional features, specifically serine protease inhibitors [16]. Antithrombin III (AT III) and heparin cofactor II (HC II) are among the most well-studied HS/heparin-binding serpins [16]. AT III prevents the activation of blood clotting proteinases through its interaction with specific blood clotting factors (thrombin, factor Xa, and factor IXa) [16]. The interaction of heparin and ATIII results in an allosteric activation, which results in a more enhanced, stable binding of serpin (AT III) and the proteinases (thrombin and factor Xa) [16]. The ATIII binds to the pentasaccharide domains within the HS/HP, which is characterized by highly sulfated moieties, including 3-*O*-sulfations [16].

Chemokines (chemotactic cytokines) are a family of secreted proteins with similar structural features, specifically, four cysteine amino acid residues in specific conserved locations [16]. Examples of chemokines that have been shown to bind to HS include platelet factor 4 (PF4), stromal cell-derived factor-1a (SDF-1a), and monocyte chemoattractant protein-1 (MCP-1) [7,16]. A pharmaceutically relevant and well-studied chemokine–HS/heparin interaction is the interaction of PF4 with heparin. The PF4–heparin interaction leads to heparin-induced thrombocytopenia (HIT), which is an adverse effect during heparin treatment, and if untreated, may lead to death. It has been shown that PF4, IL-8, and MIP-1a chemokines specifically recognize and interact with the sulfated–acetylated–sulfated domains within HS (e.g., 2-*O*-sulfated domains) [14].

## 3. Surface Plasmon Resonance

**Figure 2:**
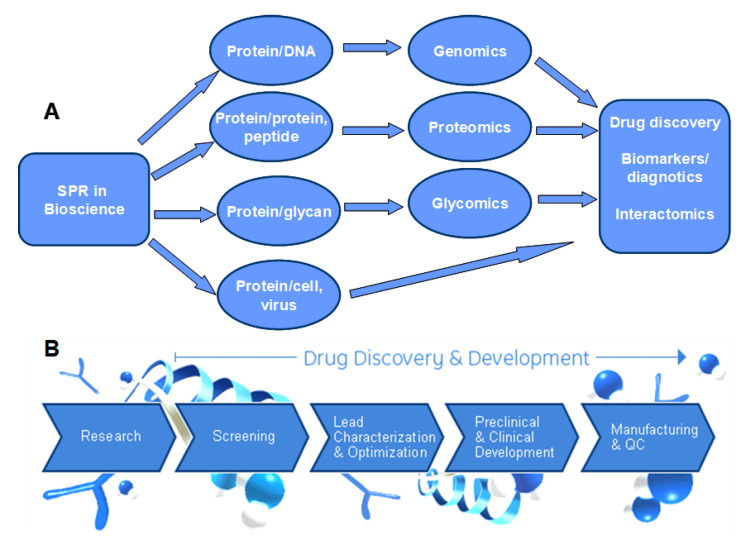
SPR has widespread applications in two major domains: (**A**) **bioscience research**, where SPR has been employed to investigate biomolecular interactions such as protein–protein, protein–DNA, polysaccharide–protein, and antibody–antigen binding; and (**B**) **drug discovery and development**, where SPR plays a crucial role in target identification and validation, high-throughput screening of potential drug candidates, lead optimization, and preclinical pharmacokinetic studies.

SPR spectroscopy has emerged as a key biophysical analysis technology in bioscience and drug discovery. The use of SPR is increasingly recognized in fundamental biological studies, health science research, drug discovery, clinical diagnosis, and environmental and biopharmaceutical process monitoring. Figure 2 shows a summary of SPR applications.

### 3.1. Principle of SPR

In SPR, a signal is generated when polarized light falls on an electrically conducting surface. This results in electron charge density waves (plasmons). This phenomenon results in a decrease in the intensity of the reflected light at the resonance angle. The reduction in the intensity of the reflected light at the resonance angle is directly proportional to the change in mass present on a sensor surface. The SPR instrument monitors changes in the resonance angle and generates data on the real-time interactions (e.g., binding affinity and binding kinetics). The data are generated as a sensorgram. In an SPR experiment, a biomolecule (ligand) is immobilized on the sensor surface. A second biomolecule (analyte) flows over the immobilized ligand. The association and dissociation of the analyte with the immobilized ligand on the sensor surface leads to a change in the refractive index near the sensor surface. This change in refractive index changes the conditions for total internal reflection of light. This results in a measurable shift in the SPR angle. This shift is recorded in real-time as an SPR sensorgram. The SPR sensorgram is a plot of the SPR signal (response units) versus time. Although SPR has high sensitivity and real-time monitoring analytical capabilities, there are some limitations, including non-specific binding (some molecules that bind non-selectively to the surface); the ligand requires immobilization on the sensor chip, which may alter the molecular conformation/orientation of the ligand.

### 3.2. Typical Workflow of SPR

**Figure 3:**
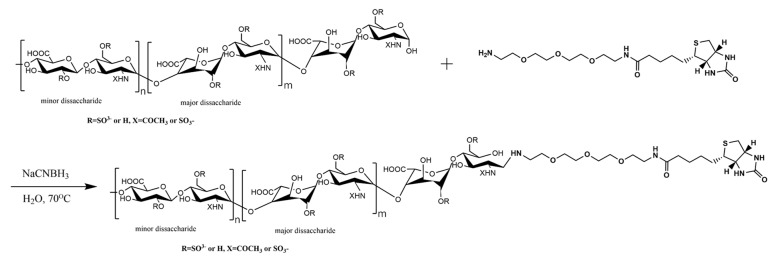
Typical reaction scheme for heparan sulfate and heparin biotinylation.

**Figure 4:**
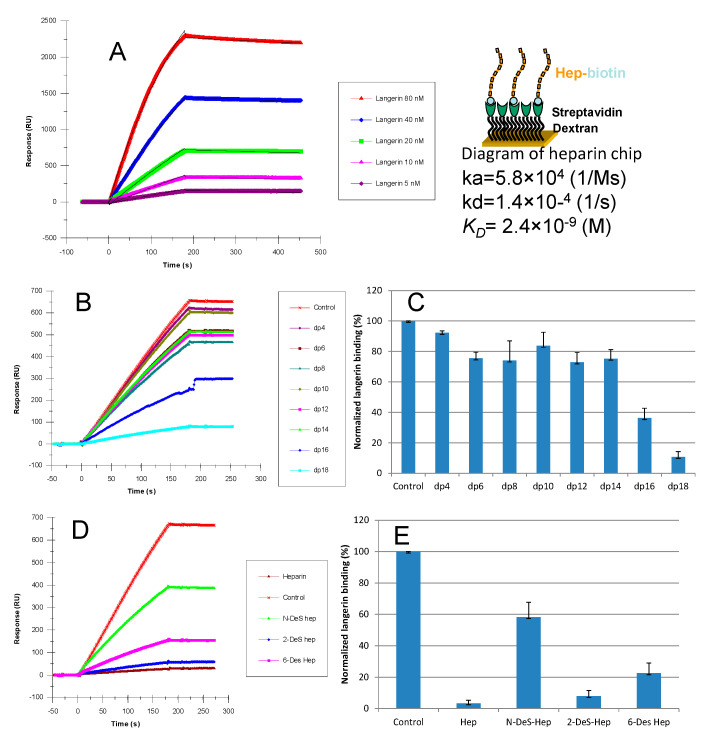
Typical SPR application for binding kinetics and structural analysis of heparin–protein interactions. (**A**) Left: SPR sensorgrams of langerin–heparin interaction; right: diagram of heparin chip and measured kinetics/affinity data for langerin–heparin interaction. Based on the sensorgrams, binding kinetics and affinity parameters (*ka*, *kd*, and *K_D_*) were calculated. (**B**) Sensorgrams of solution heparin oligosaccharides/surface heparin competition. (**C**) Bar graphs of normalized langerin binding preference to surface heparin by competing with different sizes of heparin oligosaccharides in solution, which shows the size dependence and minimum size of heparin oligosaccharide for the interaction. (**D**) Sensorgrams of solution chemical modified heparin/surface heparin competition. (**E**) Bar graphs of normalized langerin binding preference to surface heparin by competing with different chemically modified heparin in solution, which shows the sulfation dependence and sulfation preference of langerin–heparin interaction. Adapted from [52] with permission.

A typical workflow of SPR can be broadly divided into the following three steps: (1) immobilized ligand on sensor chip; (2) the measurement of interactions between ligand and the analyte; and (3) data analysis. First, the ligand is immobilized onto the sensor chip. Immobilization is achieved using various strategies, including covalent coupling and affinity capture. The covalent coupling of the ligand onto the sensor chip surface relies on the immobilization of the ligand with a reactive functional group present on the sensor chip surface. For HS/heparin–protein interaction analysis, SPR sensor chips coated with streptavidin (SA chip) have been used to immobilize biotinylated HS/heparin (Figure 3). The strategy has been successfully used for analyzing HS/HP interactions [48,49,50]. Then, the analyte is prepared in a compatible buffer and flown over the immobilized ligand. The design of SPR experiments, including the choice of sensor chips, immobilization strategy, appropriate control, and the flow rate and regeneration conditions, influences the binding kinetics [51]. The sensorgrams are used to calculate the binding affinity, kinetics, analyte concentration, and interaction specificity. Figure 4 shows a typical SPR application for binding kinetics and structural analysis on heparin–protein interactions.

## 4. SPR Applications in HS-Based Interactome

SPR has been widely used to measure the binding kinetics of the interactions of HS/heparin with numerous proteins. The following section focuses on recent developments in applications of SPR in HS/heparin–protein interactions, which are important in biomedical research [53,54].

### 4.1. SPR Application in Heparin Research

Heparin is a highly sulfated polysaccharide and is widely used as an anticoagulant drug. The first step in the industrial production of heparin is the extraction of crude heparin from porcine intestinal tissues or other animal (such as bovine or ovine) tissues. The crude heparin undergoes a series of purification and processing steps. The resultant product is unfractionated heparin (UFH). The UFH is depolymerized chemically or enzymatically to form low molecular weight heparin (LMWH), which has a longer half-life and a more predictable anticoagulant response than UFH. The purification and processing steps have been shown to impact the quality of heparin. Specifically, SPR analysis showed that autoclave sterilization process parameters caused a decrease in the biological activity of heparin [55].

Recent efforts have focused on the development of non-animal-sourced bioequivalent heparin and LMWH. A significant aspect of heparin drug development will involve evaluating the similarity with reference-listed drugs (RLD), including chemical similarity and biological equivalence. In addition, investigating the interaction of bioengineered heparin with biomolecules is essential for demonstrating the safety and efficacy of the drug. For example, the anticoagulant property of heparin is facilitated through the interaction of heparin with antithrombin III (ATIII). The comparable biomolecular interaction of bioengineered heparin with antithrombin III directly correlates with its efficacy.

**Figure 5:**
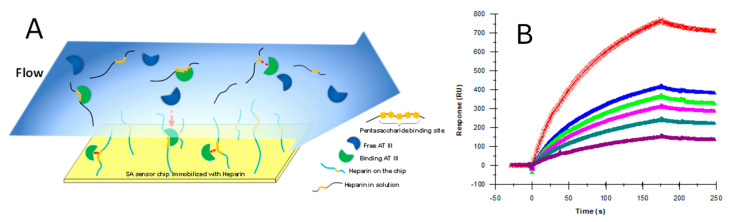
Solution competition SPR analysis of antithrombin (ATIII) and heparin interaction. (**A**) Diagram of SPR solution competition experiment for antithrombin (ATIII) binding to heparin. (**B**) SPR sensorgrams of ATIII binding to the heparin surface competing with different concentrations of heparin. The concentration of ATIII was 62.5 nM. Heparin concentrations in solution (from top to bottom) were 0, 3.13, 6.25, 12.5, 25, and 50 µg/mL, respectively. Adapted from [56] with permission.

Antithrombin III is a known serine protease inhibitor of coagulation factors, including thrombin (factor IIa). The binding of heparin to ATIII causes a conformational change in this protein. This enhances the binding affinity of ATIII with coagulation factors, including thrombin (factor IIa). The competitive SPR method (Figure 5) was successfully developed and utilized to evaluate heparin’s anticoagulant activity, and the results showed that they correlate with traditional chromogenic assays [56].

Heparin also binds to other proteins, including blood proteins. For example, platelet factor 4 (PF4 or CXCL4) is a blood protein and naturally occurring chemokine. The interaction of PF4 and heparin is associated with adverse immunological effects. Heparin administration in heparin-sensitized patients causes adverse immune reactions. The adverse immune response is due to the heparin–PF4 interaction. The interaction results in a cascading immune reaction and, in extreme cases, leads to cerebral sinus thrombosis, deep vein thrombosis, and pulmonary embolism [57]. SPR was used for the comparable analysis of the biomolecular interaction of bioengineered heparin and PF4, which is essential for mitigating potential risk [58]. The development of robust SPR techniques to evaluate heparin interactome studies will aid in developing safe and effective bioengineered heparin. However, SPR does not provide structural and biological activity analysis. Structural analysis (e.g., NMR, disaccharide, and tetrasaccharide analysis) and biological activity analysis (in vivo experiments) are required to evaluate the safety and efficacy of investigational drugs and new biological entities.

### 4.2. SPR Application in Antiviral and Antimicrobial Discovery

The interaction of viral capsid and cell-surface sulfated polysaccharides is a potential early viral attachment factor for viral pathogenesis, specifically entry of the virus into the host cell [8]. For example, in the late 1990s, Linhardt’s team discovered the role of HS in dengue virus infection: HS on the surface of host cells, as HSPGs, can act as receptors for dengue virus particles, facilitating viral entry [59]. SPR has been widely used for analysis of the binding affinity of capsid proteins of different pathogenic strains (e.g., SARS-CoV-2, dengue virus, herpes simplex virus (HSV), Zika virus, hepatitis, and MERS-CoV) with heparin [60,61,62] and other naturally occurring sulfated polysaccharides [11,60]. HS/heparin and chemically modified heparin exhibited antiviral activity [63,64,65,66,67]. Evaluating the binding kinetics of viral capsid proteins with naturally occurring and engineered sulfated polysaccharides aids in understanding the pathogenesis of the virus, as well as developing alternative new drug entities [63,68,69,70,71].

**Figure 6:**
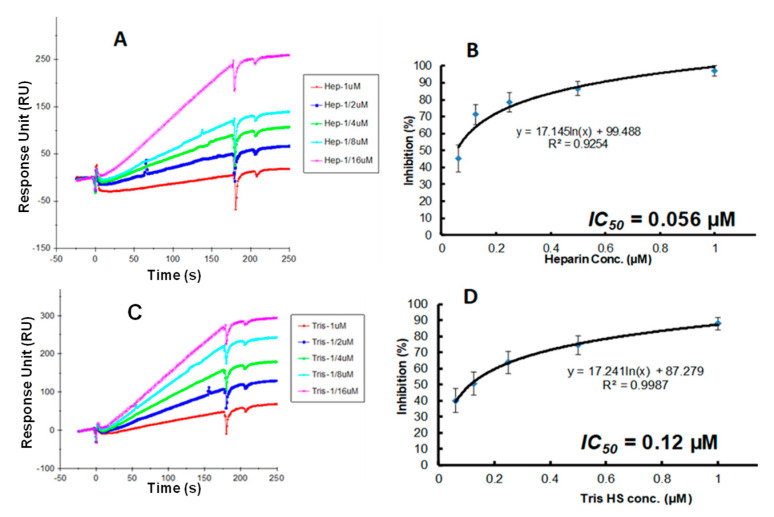
IC_50_ measurement for the inhibition of sulfated glycans on the interactions between SARS-CoV-2 S-protein and heparin using SPR. (**A**) Competition SPR sensorgrams of SARS-CoV-2 S-protein and heparin interaction inhibited by different concentrations of heparin. (**B**) Dose–response curves for IC_50_ calculation of heparin using inhibition data from competition SPR. (**C**) Competition SPR sensorgrams of SARS-CoV-2 S-protein and heparin interaction inhibited by different concentrations of tri-sulfated HS. (**D**) Dose–response curves for IC_50_ calculation of tri-sulfated HS using inhibition data from competition SPR. Data based on our previous work [62] with permission.

Glycomimetics are an emerging group of glycan-based antiviral therapeutics. Glycomimetics selectively attach to the pathogenic virus and disrupt viral interaction with host cell surface viral entry receptors (e.g., glycoproteins and proteoglycans). Broadly, two strategies exist in the screening and discovery of therapeutic glycomimetics, namely, (1) exploring naturally occurring glycans and (2) designing synthetic, semi-synthetic, and bioengineered glycans. Naturally occurring sulfated glycans (e.g., fucoidan, carrageenan, and rhamnan sulfates) from marine organisms exhibited antiviral activity against HSV (type 1 and type 2), HIV, human cytomegalovirus (HCMV), and influenza A virus (IFV) [69,70,71,72,73,74,75,76]. SPR offers a platform to screen potential glycomimetics and unlocks the potential of novel glycan-based drug products. For example, we use SPR IC_50_ measurement for the inhibition of sulfated glycans on the interactions between SARS-CoV-2 S-protein and heparin (Figure 6).

HS functions as a key mediator in microbial infections by acting as an attachment receptor for various pathogens. Its structural diversity and specific sulfation patterns enable selective binding to microbial proteins, such as bacterial adhesins, toxins, and parasitic factors, thereby facilitating pathogen entry, colonization, and immune evasion. SPR has emerged as a robust and widely used technique for characterizing the interactions of HS/heparin with various biomolecules related to microbial infection, providing critical insights into their roles in host–pathogen interactions and facilitating the discovery of novel antimicrobial agents. For example, scientists have utilized SPR to investigate the interaction of heparin/HS binding to the VacA cytotoxin, which is a virulence factor in *Helicobacter pylori* infections and type B gastritis [4]. Recently, we used SPR to characterize the kinetics of interactions between heparin and *Clostridioides difficile* (*C. difficile*) toxins (toxin A and toxin B), which are the major virulence factors for the *C. difficile* infection [77].

### 4.3. SPR Application in Neurodegenerative Diseases Research

Alzheimer’s disease (AD) is a progressive neurodegenerative disease. Alzheimer’s results in gradual memory loss and a decline in cognitive abilities, which eventually leads to dementia. In Alzheimer’s disease, the neurons in the brain cease to function and ultimately undergo neuronal cell death. The presence of extracellular amyloid-β plaques (Aβ) and intracellular neurofibrillary tangles (NFTs and tauopathy) is the hallmark feature of the AD brain. Tauopathy involves the hyperphosphorylation of microtubule-associated protein tau (MAPT), which dissociates MAPT from the microtubules and forms tau protein aggregates. The spread of tauopathy occurs through a “prion-like” infection mechanism, where abnormal tau proteins are transported from “donor cells” to “recipient cells” located in a neuroanatomically linked brain area. The transcellular transfer of tau proteins is mediated through cell-surface HS and amyloid precursor protein (APP)-mediated endocytosis/micropinocytosis. SPR studies have shown that the biomolecular interaction of tau to HS depends on the structural composition of the HS [50,78]. Specifically, the 3-*O*-sulfation domain of HS strongly increases the tau–HS interaction [50]. One therapeutic strategy to control AD progression may include inhibiting the transcellular transfer of tau proteins. A combinatorial study involving SPR, AlphaLISA, and cell-based assays has shown that heparin and fucoidan (a naturally occurring sulfated glycan) interfere with tau–HS interaction and cellular uptake of tau [79].

Parkinson’s disease (PD) is another common neurodegenerative disorder, characterized by progressive motor decline and the aggregation of α-synuclein (α-syn) protein [80]. The interaction of HS with α-syn influences the aggregation of α-syn, which contributes to Lewy body formation [81]. HSPGs play an important role in mediating cellular uptake of α-syn and Tau, which can be blocked by the addition of heparin [82]. The apparent *K_D_* for heparin in stimulating α-syn fibrillation was 0.19 μM, indicating a strong affinity for heparin and α-syn interaction [83]. SPR is useful for the measurement of binding kinetics and affinity for the interaction between HS/heparin and α-syn and the inhibitor screening for the interaction.

### 4.4. SPR Application in Cancer Research

The roles of HS and its modifying enzymes (such as heparanase and sulfatases) have emerged as a critical focus of carcinogenesis and related cancer therapeutic research due to their influence on tumor progression, metastasis, angiogenesis, and the tumor microenvironment. HS contributes to many hallmarks of cancer by modulating cell signaling and the extracellular matrix through the interactions between HS and proteins [84]. HS modulates signaling pathways such as FGF, VEGF, Wnt, and TGF-β, which are pivotal in cell growth and cancer progression. Cancer cells often alter the expression or sulfation pattern of HS chains to favor these signaling pathways, promoting proliferation [85]. In addition, HS is essential for modulating the binding of angiogenic factors like VEGF and FGF-2 to their receptors. HS functions as a co-receptor by enhancing growth factor–receptor complex formation. Tumor cells and associated stromal cells can manipulate HS structure to enhance angiogenesis, which is vital for tumor survival and expansion [86]. HS remodeling enzymes, such as heparanase and sulfatases, play a significant role in the modification of HSPGs within the tumor microenvironment. For example, heparanase cleaves HS chains, facilitating extracellular matrix degradation and releasing growth factors that support invasion and metastasis [87].

Based on the important roles of HS in cancer, several anti-tumor therapeutic strategies targeting HS and its modifying enzymes have been under investigation: (i) heparanase inhibitors (e.g., Roneparstat, a chemically modified 100% *N*-desulfated, N-reacetylated, and 25% glycol-split heparin with very low anticoagulant activity) have shown promise in preclinical clinical studies [88]; (ii) HS mimetics can competitively inhibit interactions between HS and growth factors [89]; and (iii) sulfatase inhibitors are being explored for their role in normalizing abnormal HS sulfation patterns.

In cancer research, SPR has become increasingly valuable for studying interactions between HS and a variety of proteins involved in tumor biology, as well as for therapeutic compound screening. We used SPR to study how HS/heparin interacts with various growth factors (e.g., FGF1, FGF2, VEGF, and HGF) and their receptors, chemokines, and cytokines [90,91]. This technique provides kinetic and structural data that are critical for understanding the functions of HS in cancer and for developing HS-targeted therapies. SPR has also been applied to screen small molecules or peptides that block HS–protein interactions and to test HS mimetics as antagonists of cancer signaling pathways [89].

### 4.5. SPR Application in the Research of Inflammatory Diseases

HS is involved in both acute and chronic inflammations and plays a significant role in inflammatory responses by modulating leukocyte adhesion and migration, regulating chemokine gradients, cell adhesion, and cell surface interactions [92]. HS on endothelial cells binds chemokines, creating a concentration gradient that attracts leukocytes to the area of inflammation. HS is also involved in direct adhesive interactions between endothelial cells and leukocytes, as well as between leukocytes and other cells. For example, HS is a major component of the cell surface glycocalyx and modulates interactions between pathogens and host cells during sepsis [93]. Our previous work found that sepsis leads to the release of HS fragments from the endothelium. These HS fragments, particularly those with specific sulfation patterns (N- and 2-O-sulfation), are linked to cognitive impairment in sepsis survivors [94]. We also explored the use of synthetic HS octadecasaccharide (18-mer) as a potential therapeutic agent in sepsis. This molecule (HS 18-mer) has shown promise in protecting against sepsis-related injury and improving survival in animal models [95]. SPR was a key instrument to measure the molecular interactions between HS and proteins related to sepsis, such as histone H3 and high mobility group box 1 (HMGB1), in these studies.

## 5. Conclusions

SPR has been widely used as a powerful tool in elucidating the complex interactome of HS, offering real-time, label-free insights into binding kinetics, affinity, and specificity of HS–protein interactions. Its sensitivity and adaptability have enabled the characterization of HS binding motifs, the role of sulfation patterns, and the dynamic nature of glycosaminoglycan–protein recognition. HS is a versatile molecule that participates in a wide range of biological processes, acting as a co-receptor or modulator. Its diverse interactions with proteins make it an important target for therapeutic interventions in various diseases. Applications of SPR have extended our understanding of HS-mediated biological processes while also advancing therapeutic discovery by identifying novel HS-binding partners and evaluating potential inhibitors. In parallel with the rise of AI (e.g., AlphaFold3, the next generation of protein structure prediction), advancements in data science and molecular dynamics (MD) simulation software and other analytical/biophysical tools (such as glycan arrays, NMR, LC-MS, X-ray crystallography, and cryo-EM) will enable the creation of an HS/heparin interactomics map. This map can serve as a tool to predict how HS/heparin-mediated interactions play a pivotal regulatory role in biological pathways and therapeutics. Combinatorial approaches to these techniques with SPR will provide a comprehensive insight into the glycan interactome.

## Figures and Tables

**Table 1:** Interactions of HS and heparin with varied biomolecules and pathogens.

Biomolecules/Pathogens	Examples	References
**Amyloid Proteins**	Tau protein and alpha-synuclein	[17,18]
**Cell Adhesion Molecules (CAMs)**	Cadherins, integrins, and selectins	[18,19,20,21,22,23]
**Chemokines**	Platelet factor 4, interleukin-8 (IL-8), and RANTES (CCL5)	[24,25,26]
**Growth Factors and the Receptors**	Fibroblast growth factors (FGFs), vascular endothelial growth factor (VEGF), and hepatocyte growth factor (HGF)	[27,28,29,30]
**Lipoproteins**	Low-density lipoproteins and apolipoprotein E (ApoE)	[31,32]
**Pathogens**	Viruses and viral proteins (e.g., HPV, Dengue virus, Herpes simplex virus, and HIV); bacteria (e.g., *Listeria monocytogenes*); and protozoa (e.g., malaria sporozoites)	[33,34,35,36,37,38,39,40]
**Serpins**	Antithrombin III, heparin cofactor II, and factor Xa	[41,42,43]
